# Ameliorative effect of Aconite aqueous extract on diarrhea is associated with modulation of the gut microbiota and bile acid metabolism

**DOI:** 10.3389/fphar.2023.1189971

**Published:** 2023-05-17

**Authors:** Dandan Zhang, Hao Cheng, Yuxi Zhang, Yaochuan Zhou, Jing Wu, Juan Liu, Wuwen Feng, Cheng Peng

**Affiliations:** ^1^ State Key Laboratory of Southwestern Chinese Medicine Resources, School of Pharmacy, Chengdu University of Traditional Chinese Medicine, Chengdu, China; ^2^ Key Laboratory of the Ministry of Education for Standardization of Chinese Medicine, Chengdu University of Traditional Chinese Medicine, Chengdu, China; ^3^ School of Basic Medical Sciences, Chengdu University of Traditional Chinese Medicine, Chengdu, China; ^4^ Hospital of Chengdu University of Traditional Chinese Medicine, Chengdu, China

**Keywords:** aconite aqueous extract, rhubarb, diarrhea, gut microbiota, bile acids

## Abstract

**Introduction:** Aconite is a form of traditional Chinese medicine (TCM) that has been widely used to treat diarrhea for thousands of years. However, it is not clear whether the anti-diarrhea role of aconite aqueous extract (AA) is associated with regulation of the gut microbiota or with bile acid (BA) metabolism. This study aimed to confirm whether AA exerts its anti-diarrhea effects by regulating the gut microbiota and BA metabolism.

**Methods:** The therapeutic effect of AA in a mouse model of diarrhea was measured based on analysis of body weight, fecal water content, diarrhea scores, intestinal propulsion rate, colonic pathology, and colonic immunohistochemistry. In addition, 16S rRNA high-throughput sequencing was conducted to analyze the effect of AA on the gut microbiota, and targeted metabolomics was employed to analyze the effect of AA on metabolism of BAs.

**Results:** The results showed that treatment with AA reduced fecal water content and diarrhea scores, inhibited intestinal propulsion rate and pathological changes in the colon, and increased AQP3 and AQP4 content in the colon. In addition, AA was found to be capable of regulating the gut microbiota. Effects included increasing its richness (according to the ACE and Chao1 indices); altering the gut microbiota community structure (PCA, PCoA, and NMDS); increasing the relative abundance of *norank_f_Muribaculaceae*, *Ruminococcus*, *Lachnospiraceae_NK4A136_group*, *Prevotellaceae_UCG-001*, and *norank_f_norank_o_Clostridia_UCG-014*; and decreasing the relative abundance of *Escherichia-Shigella*, *unclassified_f_Ruminococcaceae*, *Ruminococcus_torques_group*, and *Parasutterella*. More importantly, AA significantly increased fecal TCA (a primary BA) and DCA, LCA, GDCA, dehydro-LCA, and 12-keto-LCA (secondary BAs), thus restoring BA homeostasis. Moreover, AA increased the ratios of DCA/CA, DCA/TCA, and LCA/CDCA and decreased the ratios of TLCA/LCA, GLCA/LCA, and TUDCA/UDCA.

**Conclusion:** The anti-diarrhea effect of AA was associated with restoration of the gut microbiota and BA metabolism-related homeostasis. The results of this study provide insights into the application of AA and the treatment of diarrhea.

## 1 Introduction

Diarrhea is a common disease of the digestive system characterized by loose or liquid stools. Severe diarrhea can lead to disruption of water and salt metabolism or death from dehydration, and diarrhea has become a challenging problem. Currently, intestinal motility regulators (such as loperamide), intestinal secretion regulators (such as bismuth subsalicylate), antibiotics, etc., are widely used for the treatment of diarrhea (Rehydration, 2003). However, these drugs are often accompanied by many potential side effects. For example, loperamide can cause respiratory and neurological diseases, and overuse of antibiotics can lead to bacterial resistance in the body (Rehydration, 2003; Li et al., 2022). Therefore, the identification of other effective and safe drugs to treat diarrhea is necessary.

Modern pharmacological studies have shown that diarrhea is associated with gastrointestinal dysfunction and with disorders of water and salt metabolism (Wang et al., 2015; Shi et al., 2019). In recent years, the gut microbiota has become a focus of disease research (Liu et al., 2018; Mei et al., 2022). A microbiota imbalance can lead to diarrhea, which in turn promotes intestinal microecological imbalance and further aggravates diarrhea (Nie et al., 2015). Therefore, diarrhea and the gut microbiota interact with one another, and restoring the balance of the gut microbiota is an effective way to relieve diarrhea. The gut microbiota can regulate the production of metabolites, such as bile acids (BAs) and short-chain fatty acids. BAs are produced by cholesterol in the liver and converted by the gut microbiota in the gut. BAs can affect gastrointestinal peristalsis and the secretion of water and mucus in the intestine (Wei et al., 2020). BAs can also directly affect the gut microbiota through their antibacterial effects, or indirectly affect the gut microbiota through regulation of relevant receptors (Nie et al., 2015; Xie et al., 2022). Disorders of the gut microbiota and of BA metabolism have been found to occur in antibiotic-associated diarrhea (Wan et al., 2021; Xie et al., 2022), diarrhea-predominant irritable bowel syndrome (Duboc et al., 2012; Joyce and Gahan, 2017; Li et al., 2018), and inflammatory bowel disease (Hu et al., 2021). Thus, the gut microbiota and BAs play a significant role in diarrhea, and the regulation of both is an important strategy in the treatment of diarrhea.

Aconite, the processed product of *Aconitum carmichaelii* Debx. root, is a lethal poison, but is also used as a valuable and indispensable form of traditional Chinese medicine (TCM) with wide-reaching pharmacological activities, including anti-inflammation and analgesic effects; anti-tumor activity; immunoregulatory effects; hypoglycemic and hypolipidemic effects; and anti-diarrhea, anti-depressant, anti-aging, and energy metabolism effects (Singhuber et al., 2009; Wei et al., 2020; Wani et al., 2022). These pharmacological properties are attributed to various metabolites found in aconite; it is rich in alkaloids, which are known to be active compounds. The pharmacological activity of alkaloids has been demonstrated for different preparation methods in various pathological models (Singhuber et al., 2009; Wei et al., 2020; Wani et al., 2022). Aconite ethanol extract and Heishunpian (a processed product of aconite) total alkaloids have been shown to relieve diarrhea (Anwar et al., 2003; Luan and Sun, 2014; Prasad et al., 2014; Wani et al., 2022). Recent studies have shown that substances used in TCM, especially those with low bioavailability, interact with the gut microbiota after entering the intestine (Feng et al., 2021). On the one hand, the use of TCM regulates the composition of the gut microbiota and metabolism; on the other, the gut microbiota in turn metabolizes TCM compounds. The bi-direction interaction can further affect the efficacy of TCM. For aconite, many of the active ingredients show low bioavailability. For example, aconitine exhibits a low bioavailability of 4.7% (Tang et al., 2012). The low bioavailability of aconite components suggests that there may be other indirect ways for aconite to play a pharmacological role.

Although extensive research has demonstrated the anti-diarrhea effects of aconite, no study has investigated the efficacy of aconite aqueous extract (AA) in relieving diarrhea in terms of its relationship with the gut microbiota and BA metabolism. In the present study, we first explored the effects of AA in a mouse model of diarrhea. Subsequently, 16S rRNA high-throughput sequencing was used to determine the composition of the gut microbiota, and a targeted metabolomics approach was applied to detect BA metabolism in mouse feces after administration of AA. The results of this study provide a detailed explanation of the mechanism underlying the anti-diarrhea effect of AA.

## 2 Materials and methods

### 2.1 Materials

#### 2.1.1 Preparation of materials

The materials were prepared and analyzed according to our previously described methods (Liu J. et al., 2021). In brief, aconite was weighed and soaked in water at a 10-fold dilution for 30 min, and then boiled for 5 h. After filtration, water was added to achieve an 8-fold dilution, and the mixture was boiled for an additional 3 h and then filtered. The filtrate was mixed, concentrated to the required concentration, and stored at 4°C. Rhubarb was purchased from Sichuan Long Yi pharmacy (production lot number: 210501). An appropriate amount of rhubarb was weighed and mixed with water at a five-fold dilution; after soaking for 30 min, this was boiled for 15 min and filtered. The filter slag was then boiled with five-fold water for 10 min. The filtrate was combined, concentrated, and stored at 4°C. To prepare the carbon suspension, 5 g Arabic powder was mixed with 40 ml water and boiled until the solution was clear and transparent. Subsequently, 2.5 g activated carbon powder was added to the solution and this was boiled three times. After the solution had cooled, water was added to a final volume of 50 ml.

#### 2.1.2 High-performance liquid chromatography (HPLP) characterization of materials

HPLP characterization of AA was performed according to our previously described method (Liu J. et al., 2021). HPLP characterization of rhubarb aqueous extracts was performed as follows: 1 ml rhubarb aqueous solution was mixed with 4 ml methanol, centrifuged at high speed for 10 min (13000 r/min), and filtered through a 0.45-μm membrane to obtain test product solution. With reference to pharmacopeia and references, aloe emodin, rhein, emodin, chrysophanol, and physcion were accurately weighed, and methanol was added to these to prepare a reference solution containing 80 μg aloe emodin, 80 μg rhein, 80 μg emodin, 80 μg chrysophanol, and 40 μg physcion per 1 ml sample. A C18 column (250 mm × 4.6 mm, 5 μm) (Comatex, United States) was used to analyze the content of the rhubarb aqueous extract with an Agilent 1260 system HPLC instrument. The column was eluted at 25°C with the mobile phase of HPLC-grade methanol (A) and 0.1% phosphoric acid (B, 85: 15) at a flow rate of 1 ml/min; the detection wavelength was set to 254 nm and the injection volume to 10 μL. After the mixed reference solution was diluted to different concentrations, the standard curve for each reference standard in rhubarb was determined and the percentage of each component in the sample was calculated.

#### 2.1.3 Design of the animal experiment

Twenty-four specific pathogen-free (SPF) graded Kunming mice (18–22 g) were purchased from SiPeiFu (Beijing) Biotechnology Co., Ltd. (SCXK (jing) 2019–0010). The animals were housed at Chengdu University of Traditional Chinese Medicine; the temperature was maintained at 22°C ± 2°C, with a 12-h light/dark cycle, and free access to food and water was provided. After 5 days of adaptive feeding, the mice were randomly divided into three groups of eight mice: a control group (Ctrl), a model group (rhubarb 10 g/kg), and an AA group (6.2 g/kg). The model and AA groups were administered rhubarb once daily for 7 days to induce diarrhea. In addition, the AA group was administered AA 4 h after rhubarb every day for 7 days. All experiments were approved by the Animal Ethics Committee of Chengdu University of Traditional Chinese Medicine.

### 2.2 Measurement of body weight, diarrhea score, and fecal water content

During the experiment, mouse body weight was measured every day. Diarrhea scores were determined based on previous studies (Shi et al., 2019; Xu et al., 2022): 0, no diarrhea or normal stool; 1, mild diarrhea with wet and soft stools; 2, moderate diarrhea with loose stools and light perianal staining; or 3, severe diarrhea with watery stools and severe perianal staining. Fresh feces from each mouse were collected at the same time every day, weighed, and dried in an oven at 50°C for 5 h. After drying, the feces were re-weighed to calculate fecal water content.

$$Fecal\;water\;content=\frac{wet\;weight\;before\;drying-dry\;weight\;after\;drying}{wet\;weight\;before\;drying}\times 100\%$$

### 2.3 Measurement of intestinal propulsion rate

The activated carbon method was used to measure the intestinal propulsion rates in the mice. Each group was administered 0.2 ml of freshly prepared carbon suspension. After 20 min, all mice were euthanized. The intestine, from the pylorus to the cecum, was removed immediately by laparotomy and laid on a clean table without traction to measure the full length and the propulsion distance of the carbon powder. The propulsion rate of the small intestine (%) was calculated as the charcoal propulsion distance (cm)/total length of the small intestine (cm) ×100% (Lu et al., 2020).

### 2.4 Intestinal histopathology and immunohistochemistry (IHC)

Tissues from the colons of the mice were collected, cleaned with normal saline, fixed with 4% paraformaldehyde solution, dehydrated, embedded in paraffin, sliced, and stained with hematoxylin and eosin (HE). Changes in the stained tissues were observed under a digital section scanner. For IHC, colon tissues fixed with 4% paraformaldehyde solution were paraffin-embedded, sectioned, and stained according to IHC procedures. The distribution and expression of AQP3 and AQP4 in colonic tissues were observed using ImageJ image analysis software.

### 2.5 Gut microbiota analysis by high-throughput sequencing

The mice were placed into a clean cage, from which fresh feces were collected, placed in a 1.5-ml centrifuge tube, and stored in a freezer at –80°C. Fecal genomic DNA was extracted using the E. Z.N.A.^®^ Soil DNA Kit (Omega Bio-tek, Norcross, GA, United States of America). The quality of the extracted genomic DNA was assessed by 1% agarose gel electrophoresis. PCR amplification was subsequently performed on the V3-V4 variable region of the 16S rRNA gene. The upstream primer was 338F (5 ′-ACT​CCT​ACG​GGA​GGC​AGC​AGC​AG-3′), and the downstream primer was 806R (5 ′-GGACTACHVGGGTWTCTAAT-3′). Subsequently, PCR products were recovered using 2% agarose gel and purified using the AxyPrep DNA Gel Extraction Kit (Axygen Biosciences, Union City, CA, United States of America), followed by repeated 2% agarose gel electrophoresis and quantification using a Quantus™ fluorometer (Promega, United States of America). The purified PCR products were constructed using a NEXTFLEX Rapid DNA-Seq Kit and sequenced on an Illumina Miseq PE300/NovaSeq PE250 platform (Shanghai Magi Biomedical Technology Co., LTD.). The Fastp software package (version 0.19.6) was used for quality control of the double-ended original sequencing sequences, and FLASH (version 1.2.11) was used for splicing. The UPARSE software tool (version 7.1) was used to perform operational taxonomic unit (OTU) clustering with 97% similarity for the quality control concatenated sequences, and chimeras were eliminated. Sequences were evaluated against the Silva 16S rRNA gene database (v138) using the RDP classifier (version 2.11), with a confidence threshold of 70%.

### 2.6 Quantification of BAs in feces

Standard BA measures were accurately weighed and dissolved in methanol. After vortex mixing, the reserve solution was obtained. The standard solution was diluted with 50% methanol to obtain working solutions at concentrations of 1000, 500, 200, 100, 50, 20, 10, 5, 2, 1, 0.5, 0.2, and 0.1 ng/ml for further testing. After the sample was accurately weighed, the extraction solution (methanol:water = 4:1) was added, and the mixture was ground using a frozen grinder for 6 min (-10°C, 50 Hz), ultrasonicated (5°C, 40 KHz) for 30 min, and allowed to stand at −20°C for 30 min, followed by centrifugation (13,000 rcf) at 4°C for 15 min. Finally, 200 μL of supernatant was injected into an LC-MS/MS system for analysis.

UPLC-MS/MS (UHPLC-Qtrap) was used for qualitative and quantitative detection of the BA targets in the samples. Analyte compounds were separated using a Waters BEH C18 (150*2.1 mm, 1.7 μm) liquid chromatography column. A 5-μL sample was injected with a flow rate of 0.35 ml/min. The mobile phase consisted of 0.1% formic acid–water solution (solvent A) and 0.1% formic acid–acetonitrile solution (solvent B). Chromatographic separation was performed by the following gradient elution procedure: 0 min, 25% B; 10 min, 32 B%; 26 min, 75% B; 26.1 min, 100% B; 28 min, 100% B; 28.1 min, 25% B; and 33 min, 25% B at a column temperature of 40°C. Mass spectrometry data were collected using an AB SCIEX QTRAP 6500+ mass spectrometer in negative mode with an electrospray ionization (ESI) source. The optimal conditions were set as follows: curtain gas (CUR), 35; ion source Gas1 (GS1), 40; ion source gas 2 (GS2), 50. The collision gas (CAD) setting was medium, ion spray voltage (IS) was −4500V, and the temperature (TEM) was 500°C. Raw data were imported into the AB Sciex quantitative software OS, where default parameters were used to automatically identify and integrate each ion fragment and to assist in manual detection.

### 2.7 Statistical analysis

Statistical calculations were performed in GraphPad Prism (version 9.0). The results are expressed in the form of means ± standard deviation (SD). The significance of differences between the three groups was analyzed via one-way ANOVA in cases of normally distributed data. When data were not normally distributed, Kruskal–Wallis tests were used. The significance of differences between pairs of groups was analyzed via unpaired Student’s *t*-tests. *P* < 0.05 was considered to represent statistical significance.

## 3 Results

### 3.1 HPLC characterization of the materials

The five major alkaloids in AA (relative to crude aconite weight) were hypaconitine (0.83 mg/kg), mesaconitine (0.17 mg/kg), benzoylmesaconine (146.90 mg/kg), benzoylhypaconine (74.13 mg/kg), and benzoylaconine (25.56 mg/kg) (Liu J. et al., 2021). Five known components were identified in rhubarb aqueous extract through comparison of retention times with known standards: these were aloe-emodin (0.261 mg/g), rhein (1.005 mg/g), emodin (0.114 mg/g), chrysophanol (0.785 mg/g), and physcion (0.028 mg/g) (Supplementary Figure S1).

### 3.2 Effects of AA on body weight, diarrhea score, fecal water content, and intestinal propulsion rate in the mouse model of diarrhea

**FIGURE 1:**
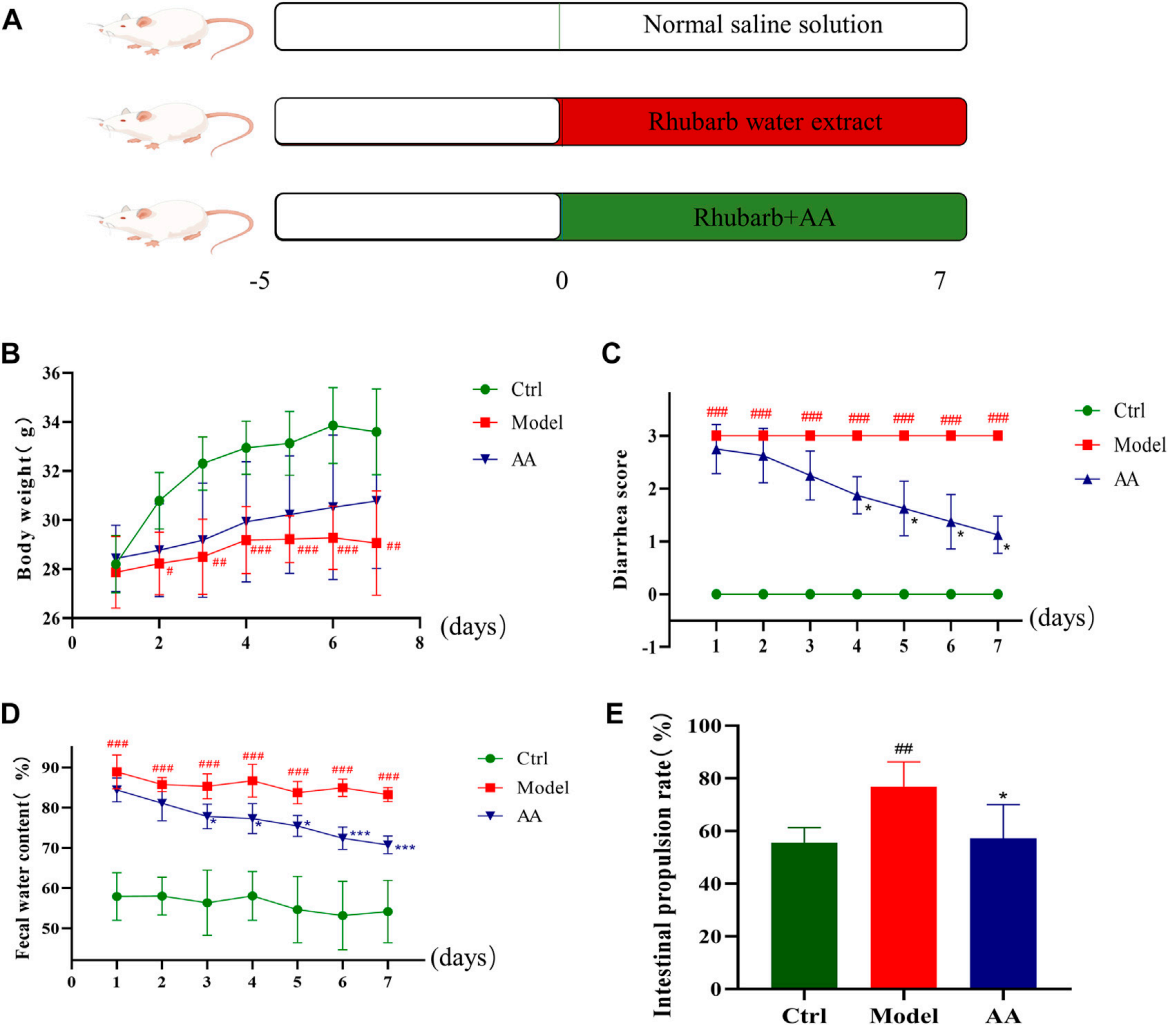
Effects of AA in the mouse model of diarrhea. **(A)** Construction of the diarrhea model and treatment of mice with AA. **(B)** Daily weight of the mice. **(C)** Diarrhea scores of the mice. **(D)** Daily fecal water content of the mice. **(E)** Intestinal propulsion rate of the mice. Data are expressed as mean ± SD, n = 8. ^#^ *p* < 0.05, ^##^ *p* < 0.01, ^###^ *p* < 0.001 compared to the control group; ^*^ *p* < 0.05, ^**^ *p* < 0.01, ^***^ *p* < 0.001 compared to the model group.

Treatments administered to each group over the full experimental period are shown in Figure 1A. As shown in Figure 1B, the body weight of the mice was significantly reduced in the model group compared with the control group. However, body weight did not differ significantly between the AA treatment group and the control group. As shown in Figures 1C, D, diarrhea scores and fecal water content were significantly increased in the model group compared to the control group (*p* < 0.001); however, both measures were significantly lower in the AA treatment group than in the model group (*p* < 0.05, *p* < 0.001). As shown in Figure 1E, the small intestinal propulsion rate of mice in the model group was increased compared with that of mice in the control group (*p* < 0.01); however, AA inhibited small intestinal propulsion rate in the mouse model of diarrhea (*p* < 0.05). These results indicate that AA regulated water metabolism and inhibited small intestine peristalsis to relieve diarrhea.

### 3.3 AA inhibited pathological changes and increased expression of AQP3 and AQP4 in the colon in the mouse model of diarrhea

**FIGURE 2:**
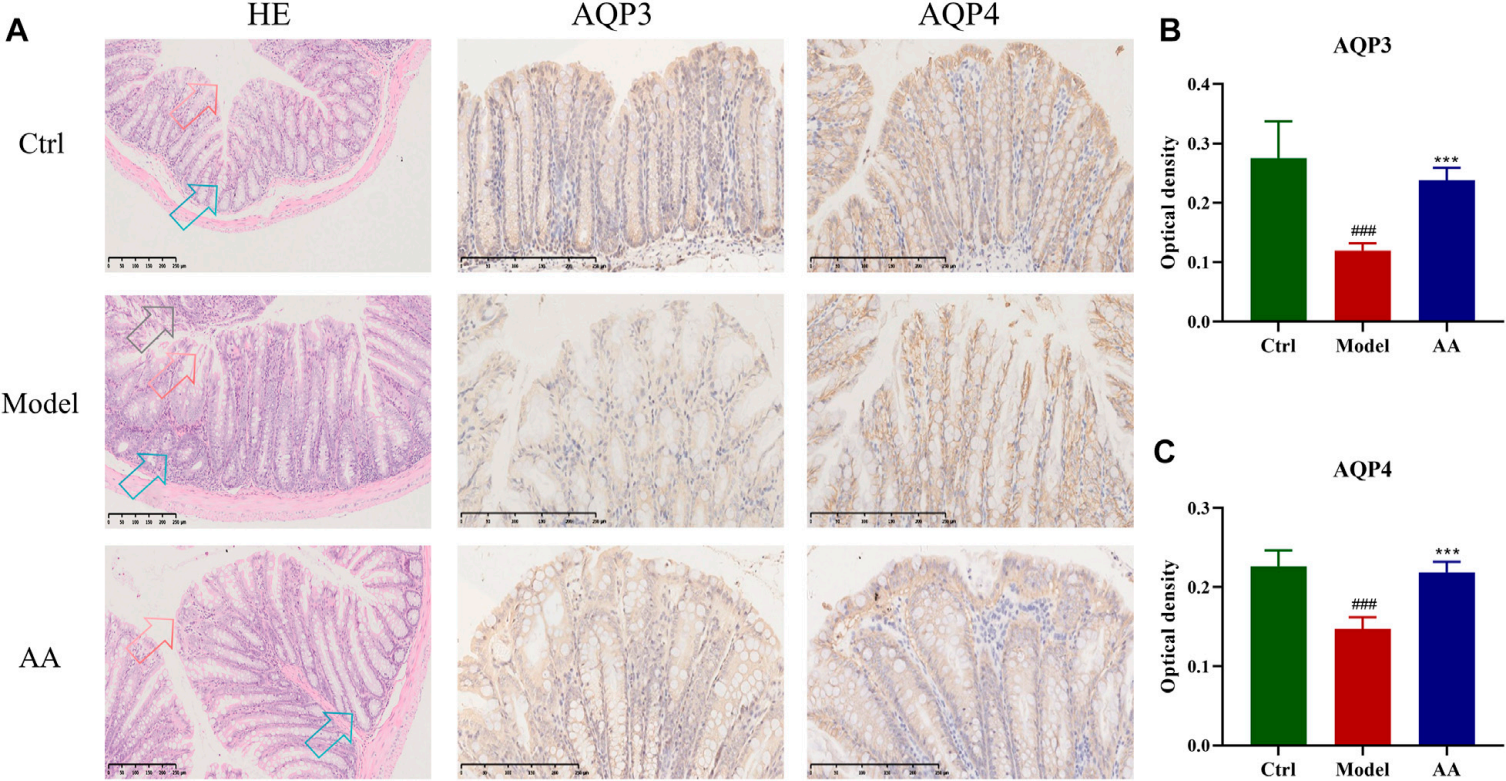
Effect of AA on the colon structure and expression of AQP3 and AQP4 in the mouse model of diarrhea (n = 6). **(A)** HE staining and IHC qualitative analysis results in colon tissue. HE staining contains three types of arrows. Red arrows, epithelial structure of the colon; blue arrows, colon glands; black arrows, inflammatory cell infiltration. **(B, C)** The expression of AQP3 and AQP4 by the IHC quantitative analysis. Data are expressed as mean ± SD, n = 6. ^###^ *p* < 0.001 compared to the control group; ^***^ *p* < 0.001 compared to the model group.

Figure 2 shows the pathological changes in the colon tissue and expression of AQP genes in each group. It can be seen that the colon epithelium of mice in the control group was intact, and the colon glands were arranged neatly, with no inflammatory cell infiltration. In contrast, the model group showed slight epithelial damage, degeneration of the colon glands, disordered goblet cell arrangement, and obvious inflammatory cell infiltration. After administration of AA, colonic epithelial cells were recovered and the colon glands were arranged neatly. IHC analysis performed to determine the effects of AA on AQP3 and AQP4 expression in the mouse model of diarrhea (Figure 2) showed that, compared with the control group, the immune reactivity of AQP3 and AQP4 in the model group mice was weaker and expression of both genes was decreased (*p* < 0.001). In the AA treatment group, expression levels of AQP3 (*p* < 0.001) and AQP4 (*p* < 0.001) were significantly increased compared to the model group (Figure 2). These findings indicate that AA can regulate water metabolism.

### 3.4 Effects of AA on the gut microbiota

**FIGURE 3:**
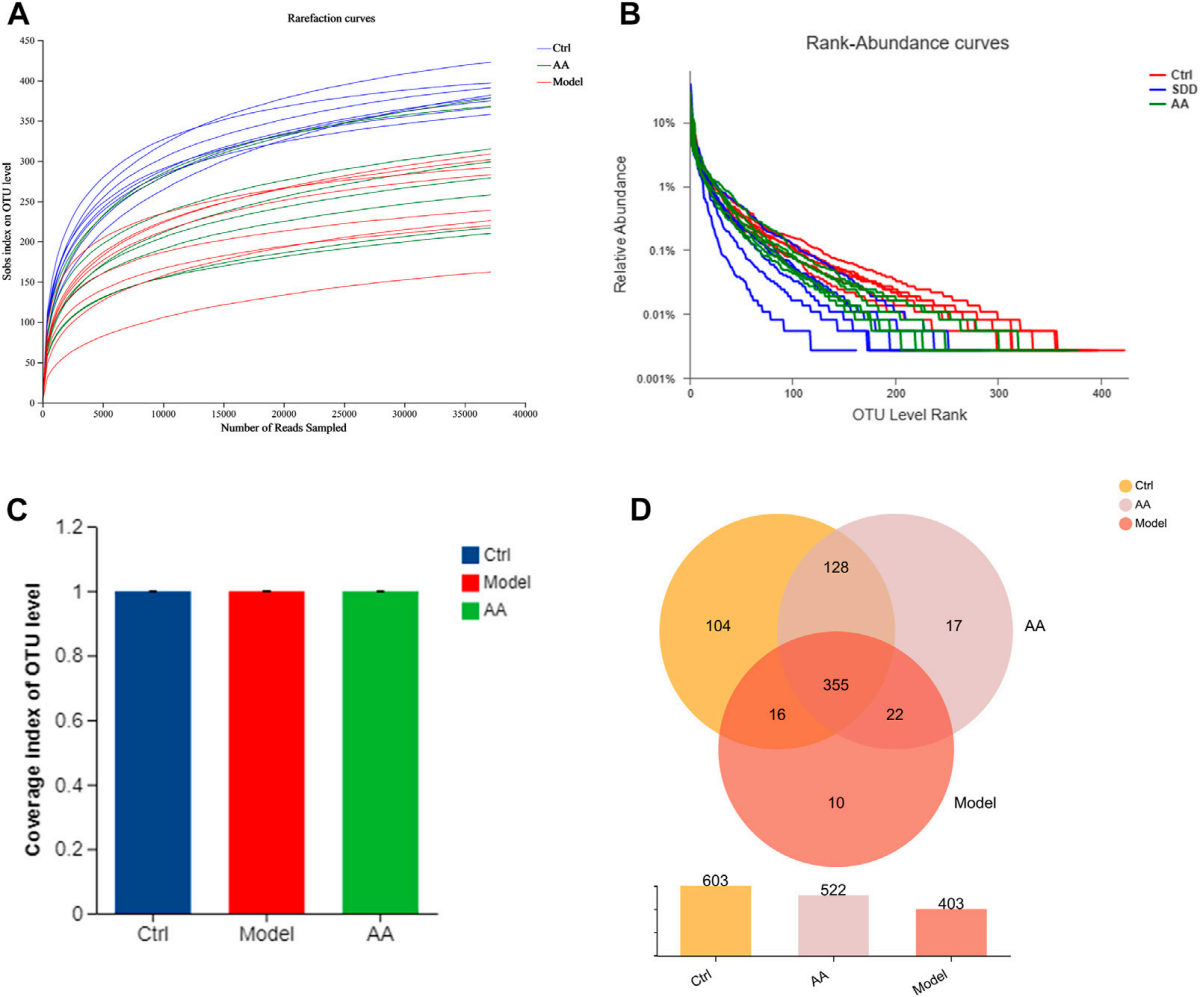
Changes in the gut microbiota in mice with diarrhea induced by rhubarb and after the AA intervention. **(A)** Rarefaction curves. **(B)** Rank abundance. **(C)** Coverage index. **(D)** Venn diagram showing OTUs in each group and the overlap between the three groups.

**FIGURE 4:**
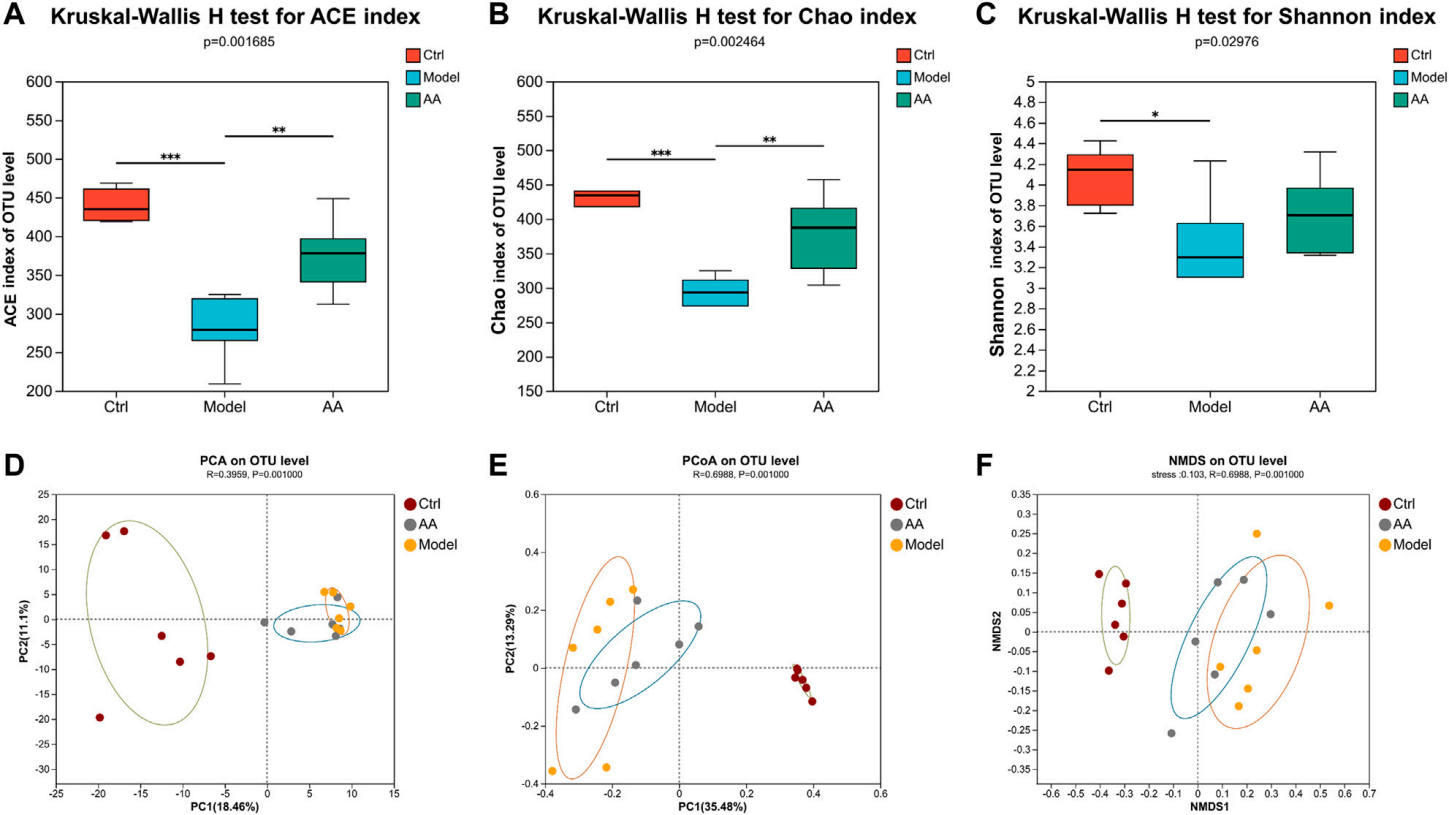
AA modulates the overall structure of gut microbiota. ACE **(A)** and Chao1 **(B)** reflect changes in community richness. Shannon **(C)** indicates changes in community diversity. Data are expressed as mean ± SD, n = 6; *p* values were calculated using the Kruskal–Wallis rank-sum test. ^*^ *p* < 0.05, ^**^ *p* < 0.01, ^***^ *p* < 0.001. **(D)** PCA. **(E)** PCoA. **(F)** NMDS. ANOSIM was used to analyze differences in PCA, PCoA, and NMDS among the three groups. Each symbol represents an individual.

To further study the mechanism of the anti-diarrhea effects of AA, 16S rRNA high-throughput sequencing was conducted to analyze the changes in the gut microbiota in the mouse model of diarrhea and to establish whether AA could effectively improve the gut microbiota of the mice. Rarefaction curves and rank abundance were plotted to visualize the biodiversity of the samples. The trends of the rarefaction curves were relatively smooth (Figure 3A), indicating that the sequencing data were reasonable and the sequencing depth was sufficient. The rank abundance curves were shallow (Figure 3B), indicating that the distribution of sequenced species was uniform. The coverage index of the sample was >99% (Figure 3C), indicating that community coverage was good and the sequencing depth was sufficient for analysis. Figure 3D shows an OTU-based Venn diagram illustrating the differences in OTU between different groups. There were 603, 403, and 522 species of bacteria in the control, model, and AA groups, respectively. Alpha diversity analysis was conducted to assess the richness and diversity of the gut microbiota: Chao1 and ACE were computed as measures of richness of microbiota, and the Shannon index was taken as a measure of diversity. As shown in Figures 4A, B, Chao1 and ACE were decreased in the model group compared with the control group (*p* < 0.001), indicating that rhubarb reduced the richness of the gut microbiota. The Chao1 and ACE indices were increased in the group administered AA, indicating that AA reversed the reduction in richness induced by diarrhea (*p* < 0.01). While the Shannon index was also decreased in the model group (*p* < 0.05) (Figure 4C), showing that diarrhea led to a reduction in gut microbiota diversity, AA administration did not result in any significant difference. Principal component analysis (PCA), principal co-ordinates analysis (PCoA), and non-metric multidimensional scaling (NMDS) were carried out to evaluate beta diversity in all samples (Figures 4D–F). The results showed that the model group could be separated from the control group, indicating that rhubarb significantly disrupted the structure of the gut microbiota. Administration of AA brought the overall structure of the gut microbiota closer to that of the control group, indicating that AA restored the microflora structure in the mouse model of diarrhea.

**FIGURE 5:**
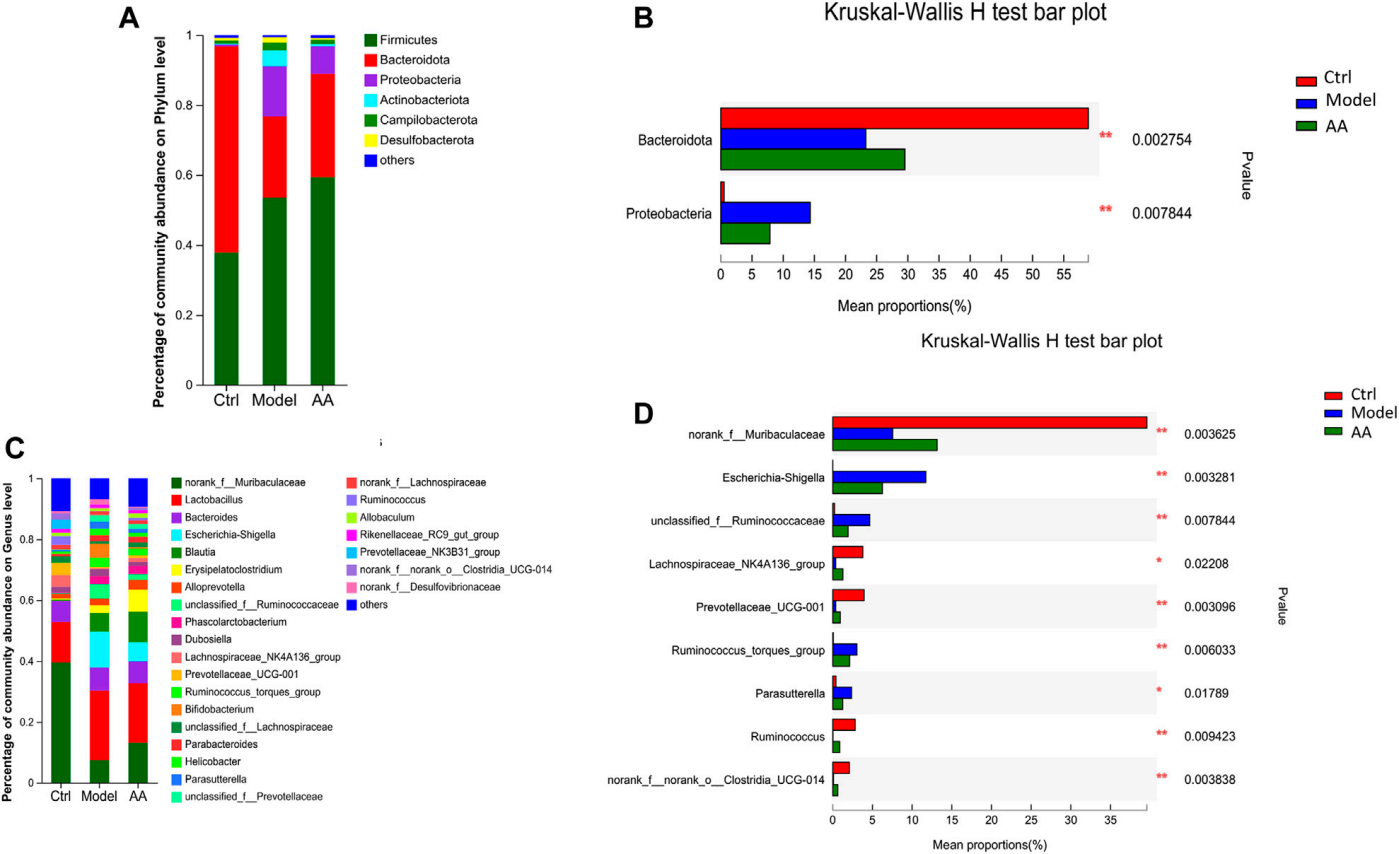
Changes in the gut microbiota in mice with diarrhea induced by rhubarb and after the AA intervention. **(A,B)** Specific differences in levels of bacteria between the three groups at the phylum level. **(C,D)** Specific differences in levels of bacteria between three groups at the genus level. Data are expressed as mean ± SD, n = 6; *p* values were calculated using Kruskal–Wallis rank-sum tests. ^*^ *p* < 0.05, ^**^ *p* < 0.01.

**FIGURE 6:**
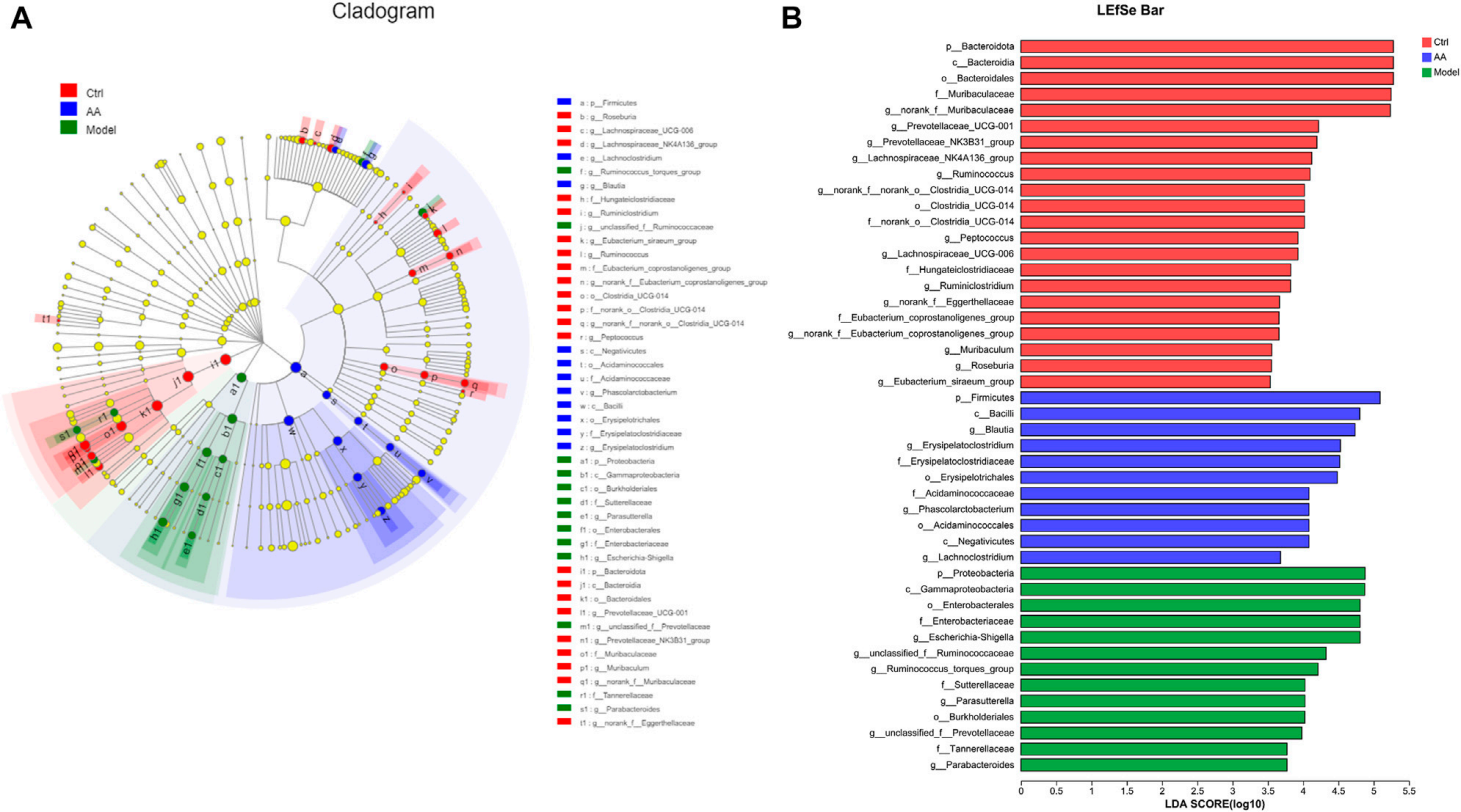
Analysis of core differential bacteria of the small intestine microbiota. **(A)** Cladogram indicating linear discriminant analysis effect size. **(B)** LEfSe analysis at the genus level (linear discriminant analysis score >3.5).

To identify the changes in strains in each group of mice and the differential bacteria that may cause and relieve diarrhea, we analyzed the gut microbiota at the phylum and genus levels. As shown in Figures 5A, B, at the phylum level, the model group mice showed a significant decrease in the abundance of Bacteroidota and a significant increase in the abundance of Proteobacteria, while the AA group showed an increase in Bacteroidota and a decrease in Proteobacteria. At the genus level as shown in Figures 5C, D, the abundance of *norank_f_Muribaculaceae*, *Lachnospiraceae_NK4A136_group*, *Prevotellaceae_UCG-001*, *Ruminococcus*, and *norank_f_norank_o_Clostridia_UCG-014* was reduced in the model group mice, while the abundance of *Escherichia-Shigella*, *unclassified_f_Ruminococcaceae*, *Ruminococcus_torques_group*, and *Parasutterella* was increased. After administration of AA, the abundance of *norank_f_Muribaculaceae*, *Lachnospiraceae_NK4A136_group*, *Prevotellaceae_UCG-001*, *Ruminococcus*, and *norank_f_ norank_o_Clostridia_UCG-014* was increased, whereas that of *Escherichia-Shigella*, *Parasutterella, unclassified_f_Ruminococcaceae*, and *Ruminococcus_torques_group* was decreased. In addition, we also identified bacteria with different levels of abundance among the different groups by LEfSe analysis and linear discriminant analysis (Figure 6). Overall, the results highlighted above revealed significant variations in the gut microbiome induced by administration of AA, showing that AA maintained intestinal homeostasis by regulating the gut microbiota.

### 3.5 Effects of AA on BA metabolism

**FIGURE 7:**
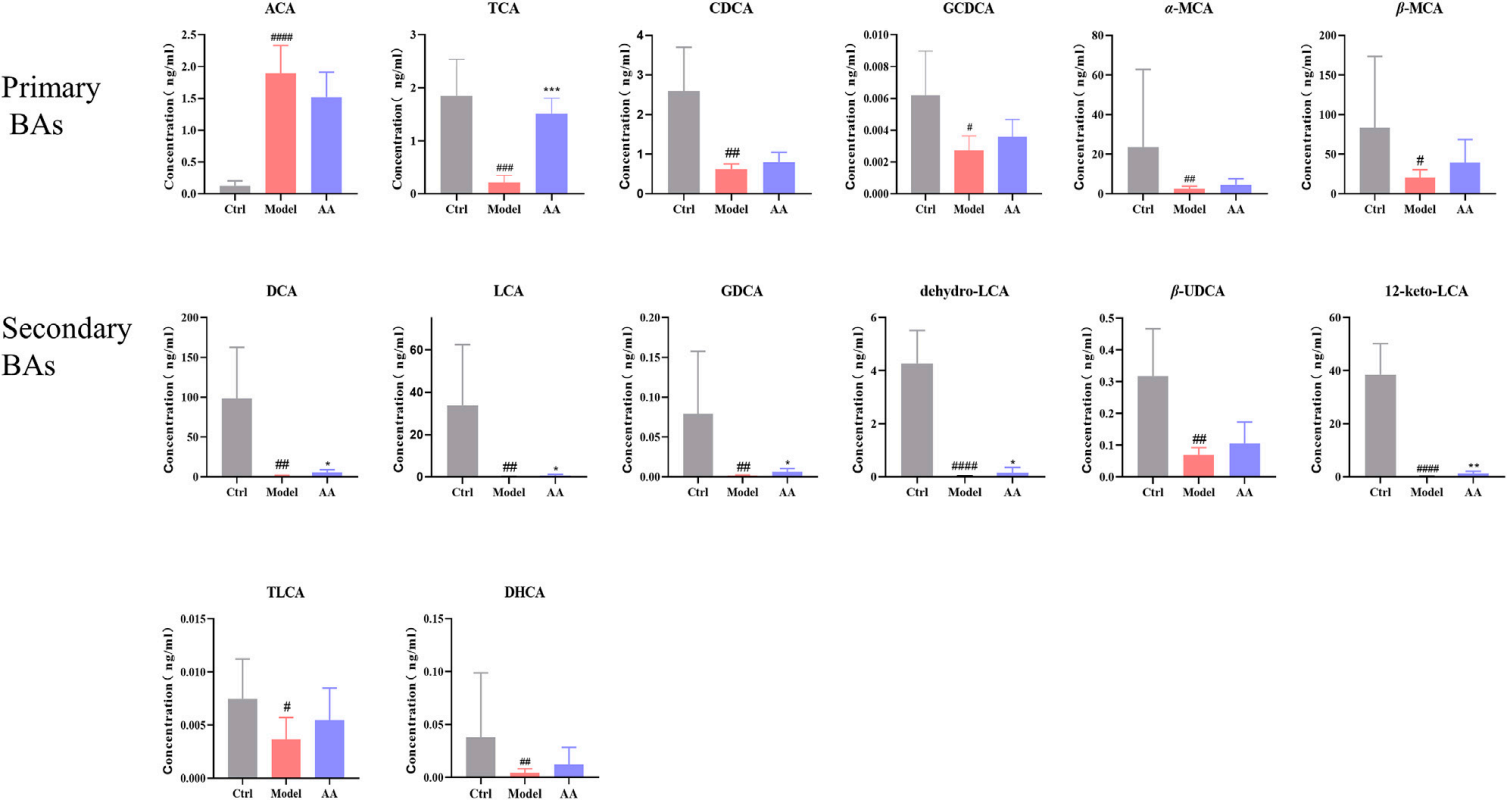
BA content in different groups of mice. ACA, apocholic acid; TCA, taurocholic acid; CDCA, chenodeoxycholic acid; GCDCA, taurochenodeoxycholic acid; *α*-MCA, alpha-muricholic acid; *β*-MCA, beta-muricholic acid; DCA, deoxycholic acid; LCA, lithocholic acid; GDCA, glycodeoxycholic acid; dehydro-LCA, dehydrolithocholic acid; *β*-UDCA, 3*β*-ursodeoxycholic acid; 12-keto-LCA, 12-ketolithocholic acid; TLCA, taurolithocholic acid; DHCA, dehydrocholic acid. Data are expressed as mean ± SD. ^#^ *p* < 0.05, ^##^ *p* < 0.01, ^###^ *p* < 0.001 compared to the control group; ^*^ *p* < 0.05, ^**^ *p* < 0.01 compared to the model group.

Metabonomics is a systems-biological approach to the study of the relationship between gut microbiota metabolism and the host. We used targeted metabolomics to analyze changes in BAs in stool samples from all groups of mice. As shown in Figure 7, among primary BAs, an increase in ACA and decreases in TCA, CDCA, GCDCA, *α*-MCA, and *β*-MCA were observed in the model group, while TCA was significantly increased upon treatment with AA. Among secondary BAs, reductions in DCA, LCA, GDCA, dehydro-LCA, *β*-UDCA, 12-keto-LCA, and TLCA were observed in the model group, while DCA, LCA, GDCA, dehydro-LCA, and 12-keto-LCA were significantly increased upon treatment with AA.

**FIGURE 8:**
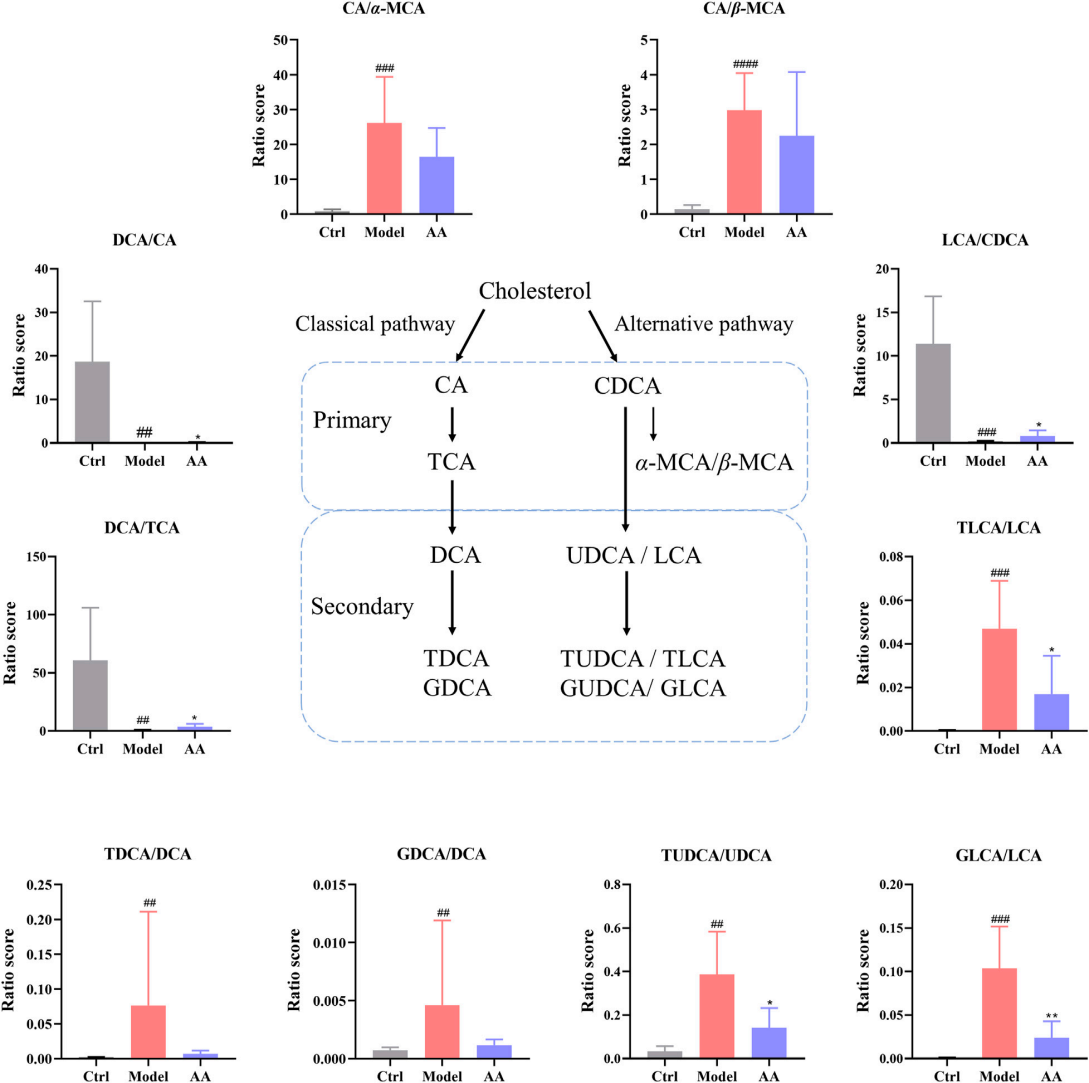
Ratios between various BAs were calculated as a measure of changes in the fecal BA pool in the different groups of mice. CA, cholic acid; *α-*MCA, alpha-muricholic acid; *β-*MCA, beta-muricholic acid; DCA, deoxycholic acid; LCA, lithocholic acid; CDCA, chenodeoxycholic acid; TCA, taurocholic acid; TDCA, taurodeoxycholate acid; GDCA, glycodeoxycholic acid; TLCA, taurolithocholic acid; GLCA, glycolithocholic acid; UDCA, ursodeoxycholic acid; TUDCA, taurodeoxycholate acid. Data are expressed as mean ± SD. ^#^ *p* < 0.05, ^##^ *p* < 0.01, ^###^ *p* < 0.001 compared to the control group; ^*^ *p* < 0.05, ^**^ *p* < 0.01 compared to the model group.

As CDCA is rapidly metabolized to *α*-MCA and *β*-MCA in the liver, we calculated the ratios of CA/*α*-MCA and CA/*β*-MCA in order to determine whether synthesis of BA in the liver shifted from the classical pathway to an alternative pathway (Hua et al., 2021). As shown in Figure 8 the ratios of CA/*α*-MCA and CA/*β*-MCA were significantly elevated in the model group, indicating that diarrhea did lead to an imbalance between the classical and alternative pathways. Although the CA/*α*-MCA and CA/*β*-MCA ratios did not change significantly after administration of AA, there was a tendency toward alleviation of the imbalance in BA metabolic pathways. In addition, since primary BAs are metabolized into secondary BAs by the gut microbiota, we conducted a statistical analysis of the ratios of DCA/CA, DCA/TCA, and LCA/CDCA in order to determine whether there was a disorder in the process of metabolism of primary BAs to secondary BAs by the gut microbiota. As shown in Figure 8, the ratios of DCA/CA, DCA/TCA, and LCA/CDCA were lowered in the model group and elevated in the AA group. In addition, in order to clarify whether changes occurred in the transformation of secondary BAs, we analyzed the ratios of TDCA/DCA, GDCA/DCA, TLCA/LCA, GLCA/LCA, and TUDCA/UDCA. As shown in Figure 8, the ratios of TDCA/DCA, GDCA/DCA, TLCA/LCA, GLCA/LCA, and TUDCA/UDCA were elevated in the model group, and the ratios of DCA/CA, DCA/TCA, and LCA/CDCA were lowered following AA. All these results indicated that abnormal BA metabolism occurred in the mouse model of diarrhea, and that AA tended to pull BA metabolism back toward its normal conditions.

### 3.6 Analysis of correlations among phenotype, gut microbiota, and BAs

**FIGURE 9:**
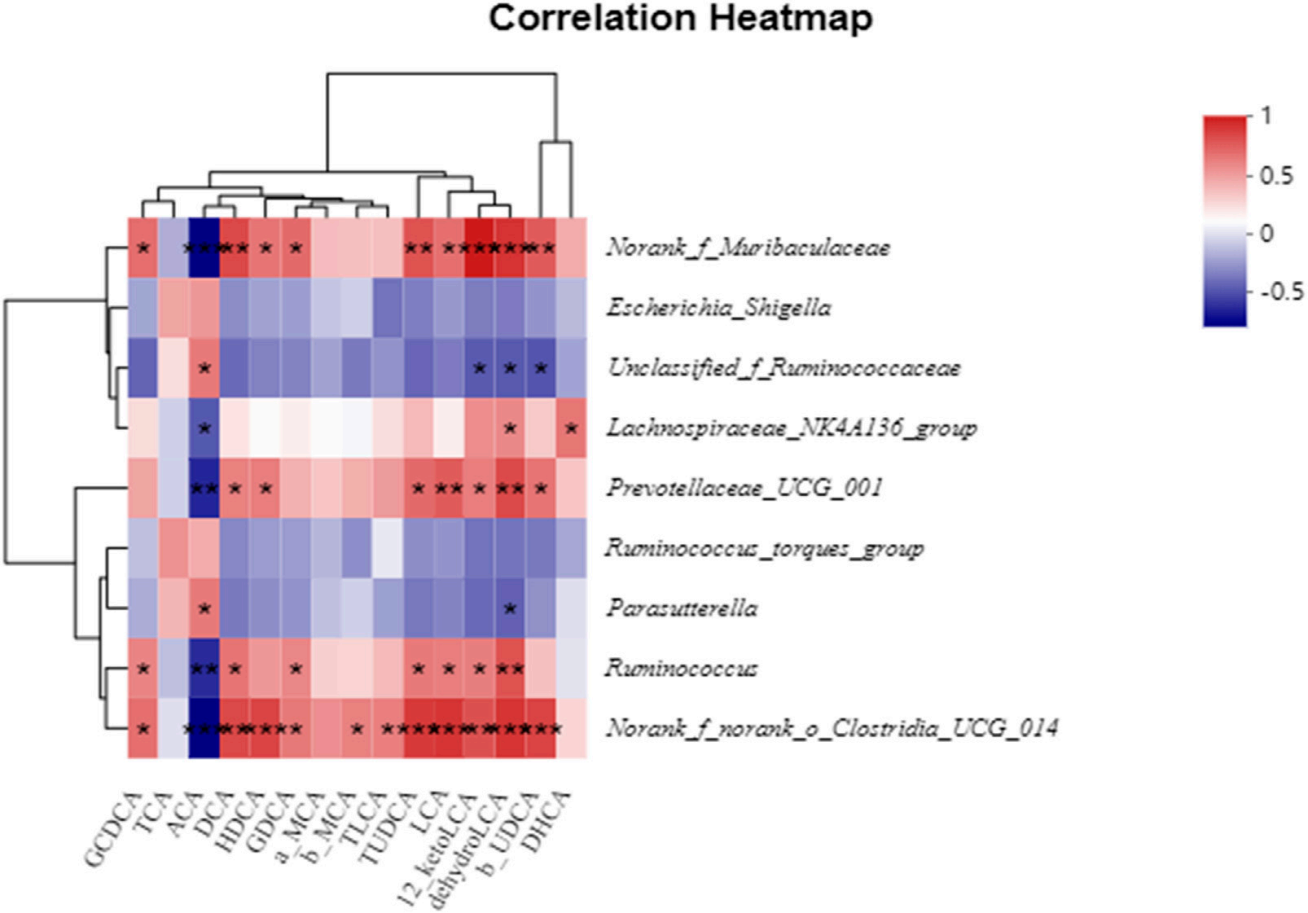
Pearson’s correlation coefficients between several significantly altered fecal BAs and genera of bacteria. ^*^ *p* < 0.05, ^**^ *p* < 0.01, ^***^ *p* < 0.001. Red, positive correlation; blue, negative correlation.

To visualize and evaluate the potential association between fecal BAs and the gut microbiota, we conducted a Pearson correlation analysis of BAs and the presence of various elements in the gut microbiota. Figure 9 illustrates the relationship between the presence of certain gut bacteria at the genus level and BAs. *Norank_f_Muribaculaceae* and *norank_f_norank_o_Clostridia_UCG-014* were positively correlated with GCDCA, DCA, GDCA, LCA, 12-keto-LCA, dehydro-LCA, and *β*-UDCA, while *Prevotellaceae_UCG-001* and *Ruminococcus* were positively correlated with DCA, LCA, 12-keto-LCA, dehydro-LCA, and *β*-UDCA. Additionally, *unclassified_f_Ruminococcaceae* was negatively correlated with 12-keto-LCA, dehydro-LCA, and *β*-UDCA.

## 4 Discussion

Diarrhea, which belongs to the “Xiexie” category under TCM theory, has become a common human intestinal disease. In order to solve the problem of diarrhea, considering the relevant therapeutic principles in TCM theory, it is vital to develop a model of diarrhea that matches the characteristics of the syndrome under the TCM view. Under the guidance of the basic theories of TCM, a variety of diarrhea models have been established over the years. Diarrhea is associated with disorders of the gut microbiota and of BA metabolism (Joyce and Gahan, 2017; Li et al., 2018; Wan et al., 2021; Xie et al., 2022). At present, folium sennae and rhubarb, categorized as “bitter-cold” based on their taste and properties under TCM theory, are the Chinese medicines most used to simulate diarrhea in animal models. Due to the antibacterial activity of rhubarb, rhubarb-induced diarrhea is associated with an imbalance of the gut microbiota (Peng et al., 2014; Xiang et al., 2020). In addition, studies have confirmed that rhubarb can relieve constipation by regulating the gut microbiota, thereby affecting BA levels. Analysis of the relevant metabolic pathways has revealed that the regulation of BAs may be the mechanism by which rhubarb exerts its purgative effect (Chen et al., 2020; Yang L. et al., 2022). The results of these studies have indicated that rhubarb-induced diarrhea is associated with modulation of BA metabolism and the gut microbiota, which is consistent with the key features of diarrhea. Therefore, rhubarb was adopted in the present study as a method of establishing a model of diarrhea. Our results showed that fecal water content and intestinal propulsion rate were increased in the mice in the model group, indicating that the diarrhea model was successful.

The gut microbiota is an important microbial community in the body and plays a central role in the maintenance of health (Zhang C. et al., 2022). Growing evidence has proven that disruption of the gut microbiota is characteristic of diarrhea (Chen et al., 2019; Breitrück et al., 2021; Wu et al., 2021; Zhen et al., 2021; Chen et al., 2022; Lu et al., 2022). In our study, 16S rRNA high-throughput sequencing was used to explore whether the structure of the gut microbiota in mice with diarrhea would be altered by administration of AA. Administration of rhubarb significantly altered the structure of the gut microbiota, which was consistent with previous research (Peng et al., 2014; Xiang et al., 2020). At the phylum level, the main change in the model group was a decrease in the level of Bacteroidota and an increase in the level of Proteobacteria. At the genus level, the levels of *norank_f_Muribaculaceae*, *Ruminococcus*, *Lachnospiraceae_NK4A136_group*, *Prevotellaceae_UCG-001*, and *norank_f_norank_o_Clostridia_UCG-014* were decreased in the model group, while the levels of *Escherichia-Shigella*, *unclassified_f_Ruminococcaceae*, *Ruminococcus_torques_group*, and *Parastterella* were increased. It is worth noting that administration of AA significantly increased the abundance of *norank_f_Muribaculaceae*, *Ruminococcus*, *Lachnospiraceae_NK4A136_group*, *Prevotellaceae_UCG-001*, and *norank_f_ norank_o_Clostridia_UCG-014* and inhibited the abundance of *Escherichia-Shigella*, *unclassified_f_Ruminococcaceae*, *Ruminococcus_torques_group*, and *Parasutterella*. *Norank_f_Muribaculaceae* has been shown to improve intestinal mucositis in mice (Li Q. et al., 2021). *Norank_f_ norank_o_Clostridia_UCG-014* can promote the proliferation of beneficial bacteria and inhibit the colonization of harmful bacteria (Liu Y. et al., 2021). *Prevotellaceae_UCG-001* is responsible for the breakdown of carbohydrates to produce short-chain fatty acids, which play an important role in improving intestinal structure (Shi et al., 2020; Zhu et al., 2021; Zhang Y. et al., 2022). *Lachnospiraceae_NK4A136_group* (belonging to the *Lachnospiraceae* family), a probiotic, produces short-chain fatty acids that inhibit pathogenic bacteria and are considered anti-inflammatory molecules. Multiple studies have identified decreased levels of *Lachnospiraceae_NK4A136_group* in the stool of mice with diarrhea, and a decrease in diarrhea symptoms is associated with increased *Lachnospiraceae_NK4A136_group* levels (Breitrück et al., 2021; Wu et al., 2021; Zhen et al., 2021; Chen et al., 2022; Lu et al., 2022). *Escherichia-Shigella* are Gram-negative, and this is the main pathogen that causes diarrhea and diarrhea-related deaths (Yan et al., 2022). *Ruminococcus_torques_group* is a bacterium whose elevated levels have been shown to damage the intestinal barrier (Huang et al., 2022). Finally, *Parasutterella*, which falls under Proteobacteria, is involved in the occurrence and development of irritable bowel syndrome (Chen et al., 2018). Therefore, these results indicated that AA exerted a clear regulatory impact on the gut microbiota of mice with diarrhea.

BAs, the final products of cholesterol breakdown, are synthesized into primary BAs in the liver and transported to the intestine, where they are metabolized by the gut microbiota into secondary BAs. BAs are regarded as endogenous laxatives, because malabsorption of BAs causes heavy enrichment of BAs in the intestinal lumen, leading to increased colon movement and even diarrhea (Xie et al., 2022). In the present study, the mice with diarrhea in the model group showed significant disorder in metabolism of BAs, which is consistent with reports that rhubarb can damage the liver (Wang et al., 2011a; Wang et al., 2011b; Li S. et al., 2021). Levels of the secondary BAs DCA, LCA, and GDCA were decreased in mice with diarrhea. Studies have confirmed the anti-inflammatory effects of secondary BAs (Jia et al., 2018; Sinha et al., 2020), and administration of AA can increase their content. We additionally evaluated the proportions of three types of metabolites as a measure of whether the entire BA metabolic chain was disturbed. CA/*α*-MCA and CA/*β*-MCA were taken to represent a shift in metabolism between the classical and alternative pathways. DCA/CA and LCA/CDCA were evaluated to assess the disruption of primary BA metabolism and secondary BA synthesis. Finally, changes in the values of TLCA/LCA, GLCA/LCA, and TUDCA/UDCA were taken to reflect imbalances in secondary BA metabolism. The results of these analyses revealed that the ratios of CA/*α*-MCA and CA/*β*-MCA were significantly increased in the mouse model of diarrhea, indicating that diarrhea induced an imbalance between the classical and alternative pathways of BA metabolism. After treatment with AA, however, while we observed no significant difference in the values of CA/*α*-MCA and CA/*β*-MCA, there was a trend toward restoration of the BA metabolic pathway. The metabolic shift between the classical and alternative pathways manifested as maladjustment of the classical pathway in the form of diarrhea. Moreover, regulation of the metabolic balance between the classical and alternative pathways may be a key point in maintaining homeostasis with respect to BAs. Additionally, we observed reductions in the values of DCA/CA, DCA/TCA, and LCA/CDCA in the model group, showing that diarrhea led to an imbalance in the conversion of primary BAs to secondary BAs. Simultaneously, treatment with AA restored these three ratios to higher values, indicating that AA can promote the conversion of primary BAs to secondary BAs. Significant changes in the ratios of primary to secondary BAs indicated variations in the components of the gut microbiota that metabolize primary BAs, suggesting that the recovery of metabolism of primary BAs into secondary BAs in the gut may also be one way to treat diarrhea. Moreover, increased ratios of TLCA/LCA, GLCA/LCA, and TUDCA/UDCA were also observed in the mouse model of diarrhea; these ratios were restored to low levels following treatment with AA. Therefore, the shift between the classical and alternative pathways of metabolism of BAs, the transformation of primary BAs to secondary BAs, and the recovery of secondary BA metabolism all indicated that AA can regulate BA metabolism to relieve diarrhea.

The liver–microbiota–metabolic axis is a future direction for research on the treatment of diarrhea. The close association of and interaction between the gut microbiota and BAs are conducive to regulation of host health. Here, the correlation between BAs and the gut microbiota at the genus level was investigated via Pearson analysis to determine whether the changes in BAs in the feces of mice with diarrhea were associated with variations in gut microbiota. The results indicated that *norank_f_Muribaculaceae* and *norank_f_norank_o_Clostridia_UCG-014* were beneficial for the formation of GCDCA, DCA, GDCA, LCA, 12-keto-LCA, dehydro-LCA, and *β*-UDCA. *Prevotellaceae_UCG-001* and *Ruminococcus* were beneficial for the formation of DCA, LCA, 12-keto-LCA, dehydro-LCA, and *β*-UDCA. However, *unclassified_f_Ruminococcaceae* inhibited the formation of 12-keto-LCA, dehydro-LCA, and *β*-UDCA. Previous studies have shown that *norank_f_Muribaculaceae* (Shao et al., 2021; Lei et al., 2022; Yang C. et al., 2022), *Parasutterella* (Ju et al., 2019; Li et al., 2019), *Ruminococcus* (Fiorucci et al., 2018; Jia et al., 2018), *Escherichia* (Fiorucci and Distrutti, 2015; Jia et al., 2018; Xie et al., 2022), *Prevotellaceae_UCG-001* (Huang et al., 2019), and *norank_f_norank_o_Clostridia_UCG-014* (belonging to the Clostridia_UCG-014 order) (Qu et al., 2022) are involved in BA metabolism. Overall, our results further suggested that AA can regulate the gut microbiota while gradually restoring disrupted BA metabolism.

AQPs, located in the top membrane of intestinal mucosal epithelial cells, are responsible for water and electrolyte transport while also protecting the integrity of the intestinal barrier (Ikarashi et al., 2016; Yde et al., 2016). Studies have shown that decreased expression of AQPs can lead to decreased water reabsorption in the colon, thereby inducing diarrhea (Xiong et al., 2018; Shi et al., 2019). Recent studies have also shown that AQPs are closely related to the gut microbiota and BA metabolism. In a diarrhea model induced by enterotoxin-producing *Escherichia coli*, treatment with *Lactobacillus plantarue* was able to produce a significantly increase in AQP3 content and regulated the gut microbiota to relieve diarrhea, and *Odoribacter* and *Bifidobacterium* were positively correlated with AQP3 levels (Yue et al., 2020). In addition, AQP4 expression was decreased in the colon of germ-free mice, but returned to normal levels after colonization by intestinal bacteria, suggesting that the gut microbiota can affect AQP4 expression in intestinal epithelial cells (Cresci et al., 2010). Furthermore, in a rat model of BA-related diarrhea caused by malabsorption of BAs, significant changes were observed in AQP3, AQP7, and AQP8 (Yde et al., 2016). In the present study, AQP3 and AQP4 content were found to be reduced in model mice, and their expression levels increased following AA treatment. In addition, AA significantly modulated the composition of the gut microbiota and BA metabolism, suggesting that the modulating effects of AA on AQPs are related to these factors. However, further studies are needed to identify the specific bacteria and BAs involved in this regulatory effect.

## 5 Conclusion

This study explored whether the ameliorative effect of AA on diarrhea is connected to the gut microbiota and to BA metabolism. Pharmacodynamically, AA showed an anti-diarrhea effect, mainly manifesting in the forms of reduced fecal water content, inhibition of small intestine progression, improvements to intestinal pathological changes, and increased AQP content. AA improved the structure of the gut microbiota, including promoting the abundance of *norank_f_Muribaculaceae*, *Ruminococcus*, *Lachnospiraceae_NK4A136_group*, *Prevotellaceae_UCG-001*, and *norank_f_norank_o_Clostridia_UCG-014* and inhibiting the abundance of *Escherichia-Shigella*, *unclassified_f_Ruminococcaceae*, *Ruminococcus_torques_group*, and *Parasutterella*. Furthermore, AA also altered BA metabolism and increased levels of the primary BA TCA and the secondary BAs DCA, LCA, GDCA, dehydro-LCA, and 12-keto-LCA to restore BA-related homeostasis. Furthermore, AA increased the ratios of DCA/CA, DCA/TCA, and LCA/CDCA, and decreased the ratios of TLCA/LCA, GLCA/LCA, and TUDCA/UDCA. Therefore, significant variations in the gut microbiota and in metabolites may be the targets of the anti-diarrhea effect of AA; this finding represents a new idea for the use of AA and for diarrhea relief.

## Data Availability

The datasets presented in this study can be found in online repositories. The names of the repository/repositories and accession number(s) can be found below: PRJNA946191 (SRA).

## Abbreviations

AA, aconite aqueous extract; ACA, apocholic acid; ANOVA, analysis of variance; BAs, bile acids; CDCA, chenodeoxycholic acid; DHCA, dehydrocholic acid; DCA, deoxycholic acid; GCDCA, taurochenodeoxycholic acid; GDCA, glycodeoxycholic acid; dehydro-LCA, dehydrolithocholic acid; α-MCA, alpha-muricholic acid; β-MCA, beta-muricholic acid; β-UDCA, 3β-ursodeoxycholic acid; IHC, immunohistochemistry; 12-keto-LCA, 12-ketolithocholic acid; LCA, lithocholic acid; NMDS, non-metric multi-dimensional scaling; PCA, principal component analysis; PCoA, principal co-ordinates analysis; SD, standard deviation; TCA, taurocholic acid; TDCA, taurodeoxycholate acid; TCM, traditional Chinese medicine; TLCA, taurolithocholic acid.
