# Supplementary material for: Ameliorative effect of Aconite aqueous extract on diarrhea is associated with modulation of the gut microbiota and bile acid metabolism
Source: Front Pharmacol. 2023 May 17;14:1189971. doi: 10.3389/fphar.2023.1189971 (PMC10229775; doi:10.3389/fphar.2023.1189971)
Supplement: Supplementary file 1 [file Image1.pdf]

Supplementary Material

Aconite aqueous extract ameliorates diarrhea via modulation of gut microbiota and bile acid metabolism

Dandan Zhang[1,2], Hao Cheng[1], Yuxi Zhang[1], Yaochuan Zhou[3], Jing Wu[1], Juan Liu[4], Wuwen Feng[1,2*], Cheng Peng[1,2*]

*** Correspondence:** Wuwen Feng: jiaoxiake-1@foxmail.com; Cheng Peng: pengchengcxy@126.com

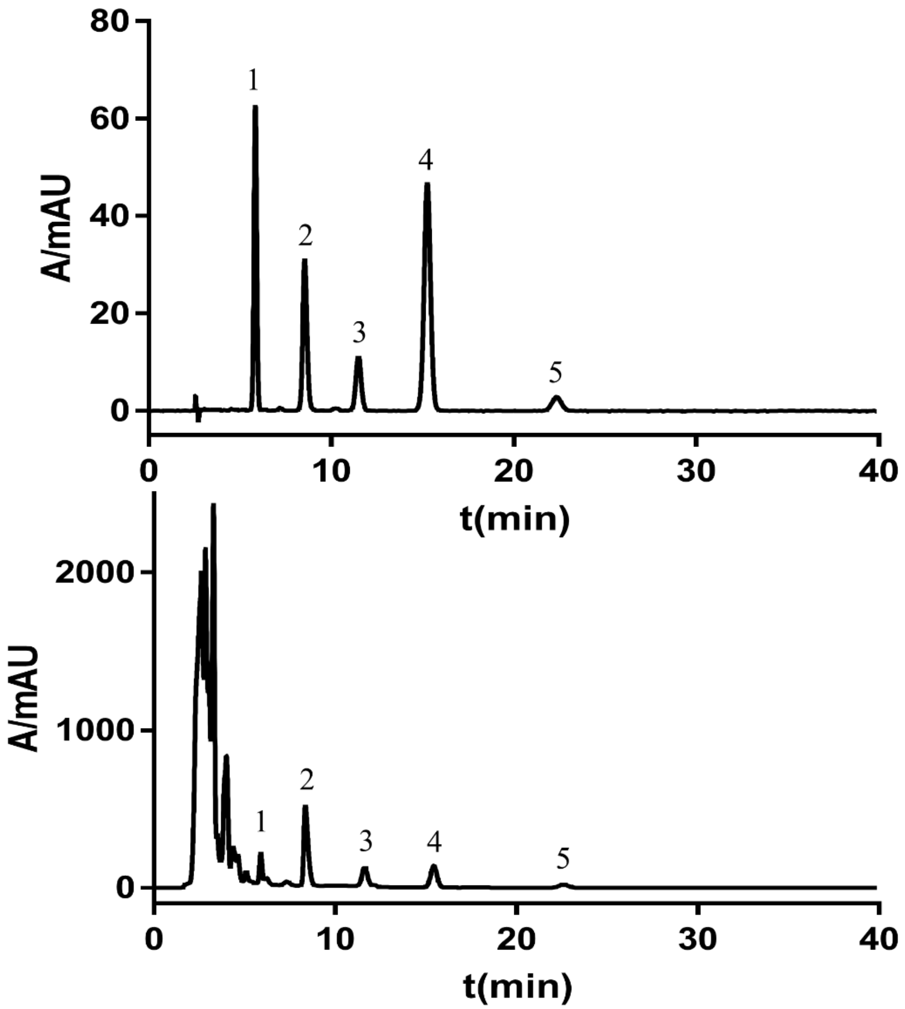

Fig. S1. HPLC chromatograms of rhubarb, rhubarb 1–5: aloe-emodin, rhein, emodin, chryshanol, physcion.
